# Hybrid Materials Formed with Green Metal-Organic Frameworks and Polystyrene as Sorbents in Dispersive Micro-Solid-Phase Extraction for Determining Personal Care Products in Micellar Cosmetics

**DOI:** 10.3390/molecules27030813

**Published:** 2022-01-26

**Authors:** Patricia I. Napolitano-Tabares, Adrián Gutiérrez-Serpa, Ana I. Jiménez-Abizanda, Francisco Jiménez-Moreno, Jorge Pasán, Verónica Pino

**Affiliations:** 1 Laboratorio de Materiales para Análisis Químicos (MAT4LL), Departamento de Química, Unidad Departamental de Química Analítica, Universidad de La Laguna (ULL), La Laguna, 38206 Tenerife, Spain; pnapolit@ull.edu.es (P.I.N.-T.); agutiers@ull.edu.es (A.G.-S.); aijimene@ull.edu.es (A.I.J.-A.); fjimenez@ull.edu.es (F.J.-M.); 2 Unidad de Investigación de Bioanalítica y Medioambiente, Instituto Universitario de Enfermedades Tropicales y Salud Pública de Canarias, Universidad de La Laguna (ULL), 38206 Tenerife, Spain; 3 Laboratorio de Materiales para Análisis Químicos (MAT4LL), Departamento de Química, Unidad Departamental de Química Inorgánica, Universidad de La Laguna (ULL), La Laguna, 38206 Tenerife, Spain

**Keywords:** metal-organic frameworks, polystyrene, polymeric hybrid materials, dispersive micro-solid-phase extraction, personal care products

## Abstract

Hybrid materials based on polystyrene (PS) and green metal-organic frameworks (MOFs) were synthesized, characterized, and evaluated as potential sorbents in dispersive micro-solid-phase extraction (µ-dSPE). Among the resulting materials, the hybrid PS/DUT-67(Zr) was selected as the adequate extraction material for the monitoring of six personal care products in micellar cosmetic samples, combining the µ-dSPE method with ultra-high performance liquid chromatography (UHPLC) coupled to ultraviolet/visible detection (UV/Vis). Univariate studies and a factorial design were performed in the optimization of the microextraction procedure. The compromise optimum extraction conditions included 20 mg of PS/DUT-67(Zr) for 10 mL of sample, 2 min of extraction time, and two desorption steps using 100 µL of acetonitrile and 5 min assisted by vortex in each one. The validated μ-dSPE-UHPLC-UV/Vis method presented limits of detection and quantification down to 3.00 and 10.0 μg·L^−1^, respectively. The inter-day precision values were lower than 23.5 and 21.2% for concentration levels of 75 μg·L^−1^ and 650 μg·L^−1^, respectively. The hydrophobicity of the resulting PS/DUT-67(Zr) material was crucial for the improvement of its extraction capacity in comparison with its unitary components, showing the advantages of combining MOFs with other materials, getting new sorbents with interesting properties.

## 1. Introduction

Sample preparation remains as a crucial step nowadays to ensure: (i) proper elimination of the interferences present in a sample, (ii) the preconcentration of target analytes present at trace levels in the sample, and (iii) the compatibility with the further analytical instruments [1], particularly for complex samples and despite advances in analytical instrumentation. It is also fair to recognize that conventional extraction techniques, such as the widely used and well-known solid-phase extraction (SPE) and liquid–liquid extraction (LLE) techniques, require improvements or alternatives to reach current green expectations. Within these new trends, improved and alternative analytical sample preparation methods have been proposed to overcome limitations of SPE and LLE [2].

Miniaturized and automated procedures have attracted significant attention in this sense. This has led to the introduction of different microextraction methods, where the extraction material (sorbent or solvent) has gained a bigger relevance due to its major influence in the process [3]. Thus, their establishment has also involved an unavoidable convergence between material science and analytical chemistry through the incorporation of new smart materials, such as ionic liquids (ILs) and their derivatives [4,5], nanoparticles (NPs) [6], deep eutectic solvents (DESs) [7], covalent-organic frameworks (COFs) [8], molecular imprinted polymers (MIPs) [9], and metal-organic frameworks (MOFs) [10,11].

Among above mentioned materials, MOFs are of special interest. These polymeric crystalline three-dimensional structures are constituted by two types of secondary building units (SBUs), metallic ions (or clusters), and organic ligands (linkers), connected through coordination bonds. These highly ordered frameworks are particularly characterized by their high porosity, impressive surface areas, and outstanding synthetic tuneability, thus allowing an endless number of possible metal-ligand combinations, together with feasible post-synthetic modifications [12]. Given these properties, it is not surprising the number of studies incorporating MOFs as stationary phases in chromatography [13,14], or as extraction sorbents in different miniaturized solid-phase extraction techniques, such as micro SPE (μ-SPE), dispersive miniaturized SPE (μ-dSPE), and solid-phase microextraction (SPME), among many other variants [10,15].

Nevertheless, despite their outstanding potential, MOFs are not an exception and exhibit a few limitations that hinder their full exploitation. For example, several MOFs do not present high chemical stability in water, representing the biggest challenging shortcoming for their analytical applications, and in other cases, the strong interactions between MOFs and analytes complicate the further release of these compounds during the desorption step [16,17]. With the purpose of improving the hydro-stability of MOFs, and even their thermal and structural stability, efforts have shifted, on one hand, to the synthesis of water-stable MOFs [18,19] and, more recently, to the preparation of MOFs-based composites [16,20].

Given the interest of composites, researchers have started to merge MOF crystals with other materials, attempting to mitigate the weakest points of their single neat components through the combination of their best properties. Thus, different MOF-based composites and hybrid materials have been reported by incorporating them into inorganic materials (mesoporous silica) [21,22], polymers [23,24], metal oxide particles [25,26], and carbonaceous materials such as graphene oxide, carbon nanotubes, etc., [25,27]. This advance has made possible the design of sorbent materials with novel physicochemical features that lead to an enhancement of MOFs extraction performance by giving them higher structural support, by protecting their pores, or even by providing them with hydrophobicity and magnetic properties. Among these composites using polymers, those mostly used include chitosan [24], alginate [23], methacrylate derivatives [28], or polyvinylidene fluoride [29]. Composites made by MOFs and polystyrene (PS) have also been reported intending, from an inorganic chemistry point of view, the development of core-shell structures, mainly having PS beads as core, and MOFs coatings as shell [30,31,32,33]. However, the analytical application of this type of composite has been limited to two analytical applications using the MOF MIL-53(Al) [30] and ZIF-8 [31].

Herein, we report the preparation and characterization of two novel hybrid materials based on the blending of PS and two MOFs, specifically DUT-67(Zr) and CIM-80(Al), given their green synthetic conditions and the absence of dimethylformamide (DMF) in their preparation, ensuring compromise with green analytical chemistry requirements. The synergistic effect of the neat components was studied in a preliminary evaluation of both hybrid materials, PS/DUT-67(Zr) and PS/CIM-80(Al), as potential sorbent materials in μ-dSPE. Finally, a μ-dSPE method, in combination with ultra-high performance liquid chromatography (UHPLC) coupled to ultraviolet/visible detection (UV/Vis), is proposed for the monitoring of six personal care products (PCPs) in cosmetic samples by using PS/DUT-67(Zr) as extraction material.

## 2. Experimental Section

### 2.1. Standards, Reagents, Materials, and Samples

Six PCPs, including four preservatives and two UV filters, were selected for this study. Methylparaben (MPB), ethylparaben (EPB), and propylparaben (PPB), were acquired from Dr Ehrenstorfer GmbH (Aungsburg, Germany). Benzylparaben (BzPB), benzophenone (BP), and benzophenone-3 (BP3), were purchased from Sigma-Aldrich (Steinheim, Germany). All these standards were obtained as solid products with purities higher than 98%. Stock solutions of each analyte were prepared in acetonitrile (ACN) LC-MS grade, supplied by VWR International (Barcelona, Spain), with concentrations between 1000 and 4500 mg·L^−1^, depending on the specific compound. Intermediate standard solutions, containing all the mentioned analytes, were prepared by the dilution of the stock solutions in ACN and stored at 4 °C. Working standard solutions were daily prepared at different concentrations, within the range 10 to 6000 µg·L^−1^, depending on the specific study. Table 1 shows the structures and several chemical properties of the PCPs studied.

Zirconium (IV) oxychloride octahydrate (98%), 2,5-thiophenedicarboxylic acid (H_2_TDC, 99%), aluminum nitrate nonahydrate (98%), mesaconic acid (99%), urea (99%), and sodium acetate anhydrous (≥99%), were employed in the synthesis of the MOFs DUT-67(Zr) and CIM-80(Al). Azobisisobutyronitrile (AIBN, 98%), polyvinylpyrrolidone (PVP, average molar wt. 10000), and styrene (≥99%), were used in the preparation of PS spheres and the proposed MOFs-based materials. All these reagents were supplied by Sigma-Aldrich. Ethanol and acetic acid were purchased from Merck KGaA (Darmstadt, Germany) and utilized in the synthesis and for the cleaning procedures of the different materials. Methanol LC-MS Chromasolv^TM^ was acquired from Honeywell (Charlotte, NC, USA) and used in the optimization of μ-dSPE method.

Liquid chromatography mobile phases were prepared with ACN LC-MS grade and ultrapure Milli-Q water obtained by a water purification system A10 MilliPore (Watford, UK). The mobile phases were always filtered using Durapore^®^ polyvinylidene fluoride (PVDF) membrane filters of 0.22 μm acquired from Merck KGaA.

Stainless steel autoclaves and Teflon solvothermal reactors, purchased from Parr Instrument Company (Moline, IL, USA), were used for the synthesis of CIM-80(Al) MOF. The synthesis of DUT-67(Zr) did not require solvothermal reactors but glass equipment for the reflux.

Pyrex^®^ centrifuge tubes (Staffordshire, UK), with a volume of 15 mL and dimensions of 9.5 × 2 cm, PVDF (0.2 μm) Whatman^TM^ syringe filters, supplied by GE Healthcare (Buckinghamshire, UK), and a 2 mL Fortuna Optima^®^ glass syringe, acquired from Sigma-Aldrich, were all employed in the μ-dSPE procedure.

Three commercial micellar cosmetic samples were selected and analyzed. They were purchased in local stores. All of them were classified as paraben-free products. Prior to their analysis, adequate aliquots of the samples were diluted up to 100 mL, and the pH value was measured.

### 2.2. Instrumentation

A UF30 oven, supplied by Memmert GmbH + Co. KG (Schwabach, Germany), was used for the synthesis of CIM-80(Al) MOF. A hot-plate magnetic stirrer and a Sensorterm II electronic contact thermometer from JP Selecta^®^ (Barcelona, Spain) were employed for the preparation of DUT-67(Zr) MOF and for the MOFs-based hybrid materials.

A vortex mixer, and a centrifuge model 5702, acquired from Velp^®^ Scientifica (Usmate, Italy) and Eppendorf^TM^ (Hamburg, Germany), respectively, were utilized in the μ-dSPE procedure.

The phase identification of the prepared MOFs and hybrid materials was performed with an Empyrean diffractometer from Malvern Panalytical (Almelo, The Netherlands), operating with Bragg-Brentano geometry. Data collection was accomplished using Cu-Kα radiation (λ = 1.5418 Å) over the angular range from 5.01° to 79.99°, with a total exposure time of 10 min (approximately).

Fourier transform infrared (FT-IR) spectra (500–4000 cm^−1^) for the characterization of the PS microspheres, were obtained with an IFS 66/S spectrometer from Bruker (Billerica, MA, USA).

Nitrogen adsorption isotherms of the PS spheres, the neat DUT-67(Zr) and CIM-80(Al) MOFs, and the resulting hybrid materials, were measured with a surface area analyzer Gemini V 2365, supplied by Micromeritics (Norcross, GA, USA), at 77 K, in the range of 0.006 ≤ P/P_0_ ≤ 1.00. The degas temperature was 363 K. The Brunauer, Emmett and Teller (BET) method was used to calculate the surface area. A Discovery SDT 650 simultaneous thermal analyzer from TA Instruments (New Castle, DE, USA) was also employed for the thermogravimetric and differential thermal analyses (TG/DTA) of PS/DUT-67(Zr) material.

The morphological characteristics of the synthesized materials were observed through a JSM6300 and a JSM6400 scanning electron microscopes (SEMs), both from JEOL (Tokyo, Japan).

Chromatographic analysis was performed using a UHPLC 1260 Infinity Series system from Agilent Technologies (Santa Clara, USA). The instrument was equipped with a quaternary pump and a Rheodyne 7725i injection valve with a loop of 20 μL. A UV-Vis ProStar 325 LC detector, supplied by Varian (Palo Alto, CA, USA), was used.

The separation of the PCPs was accomplished using an EC-C18 InfinityLab PoroShell column (50 mm × 4.6 mm × 2.7 μm) from Agilent Technologies at 25 °C, and a binary mobile phase constituted by ACN and ultrapure Milli-Q water (with a 0.1% of acetic acid), under a constant flow rate of 0.5 mL·min^−1^. The elution gradient employed to achieve the optimal separation of the analytes started at 50% of ACN, holding it for 1 min. Next, it was gradually increased to 90% of ACN in 8 min and, finally, to 100% in 1 min. The detection wavelength was set at 254 nm. Figure S1 shows a representative chromatogram obtained by direct injection of a standard solution at a concentration level of 100 μg·L^−1^.

### 2.3. Procedures

#### 2.3.1. Synthesis of MOFs

The CIM-80(Al) MOF was synthesized according to the hydrothermal method described by Rocío-Bautista et al. [34] with some modifications. Briefly, a mixture of mesaconic acid (2 mmol, 260 mg), aluminum nitrate nonahydrate (2 mmol, 750 mg), and urea (1 mmol, 60 mg), was dissolved in 30 mL of deionized water, under constant stirring. Subsequently, the mixture was transferred to a 45 mL Teflon-lined stainless-steel autoclave and heated at 150 °C for 12 h. The resulting white crystals were collected by centrifugation (1228× *g* for 5 min), washed several times with deionized water, and dried at 105 °C.

The DUT-67(Zr) MOF was prepared following the procedure proposed by Reinsch et al. [35]. Briefly, a mixture of H_2_TDC (6.73 mmol, 1166 mg), and zirconium (IV) oxychloride octahydrate (10.18 mmol, 3278 mg), was dissolved in 25.4 mL of deionized water and 25.4 mL of concentrated acetic acid. Afterwards, the mixture was transferred to a round bottom flask and heated at 95 °C for 24 h, under constant stirring. The resulting product was gathered by centrifugation (1228× *g* for 5 min), washed with a 0.1 M sodium acetate solution and deionized water (three times with each solvent), and dried at 105 °C.

Both MOFs were activated under vacuum conditions at 150 °C for 24 h, before their utilization.

#### 2.3.2. Synthesis of Hybrid Materials Based on PS and MOFs

PS/MOF materials were prepared following dispersion polymerization strategies already reported [32,36]. Briefly, PVP (600 mg) was dissolved in 40 mL of an ethanol/water mixture (80:20, *v*/*v*). Then, the selected MOF (600 mg) was dispersed in such medium by sonication for 20 min, and then styrene (2.2 mL) was added. The resulting mixture was purged with nitrogen for 30 min under constant stirring. Thereafter, AIBN (50 mg) was dissolved in 10 mL of ethanol/water (80:20, *v*/*v*) and added. The polymerization was accomplished at 70 °C for 24 h, under an inert atmosphere. The obtained white product was collected by centrifugation (1228× *g* for 15 min), washed several times with an ethanol/water mixture (80:20, *v*/*v*), and air dried. The obtained PS/MOF materials were also activated under vacuum conditions at 90 °C for 24 h.

PS microspheres were also prepared following the procedure described above, but in absence of MOFs.

#### 2.3.3. Dispersive Micro-Solid-Phase Extraction (μ-dSPE) Method

**Figure 1:**
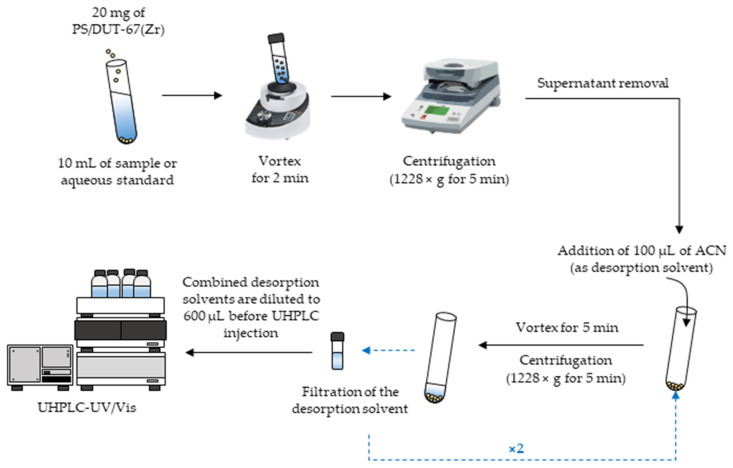
Scheme of the μ-dSPE procedure performed under optimum conditions.

The μ-dSPE procedure was optimized using the PS/DUT-67(Zr) material as the adequate extraction sorbent. Under optimum μ-dSPE conditions, 20 mg of PS/DUT-67(Zr) was added to 10 mL of sample (or aqueous standard solution) in a 15 mL Pyrex^®^ centrifuge tube. The material was dispersed through vortex agitation for 2 min and then collected by centrifugation (1228× *g* for 5 min). Once the aqueous supernatant was removed, the first desorption step was accomplished using 100 μL of ACN under vortex agitation for 5 min. Afterwards, the tube was subjected to centrifugation (1228× *g* for 5 min) and the supernatant was filtered through 0.2 μm PVDF syringe filters. This desorption procedure was repeated a second time following the same conditions. The combined eluates were diluted to 600 μL with ultrapure Milli-Q water, followed by injection in the UHPLC-UV/Vis. Figure 1 shows a scheme of the optimum μ-dSPE procedure.

## 3. Results and Discussion

### 3.1. Characterization of the PS/MOF Materials

**Figure 2:**
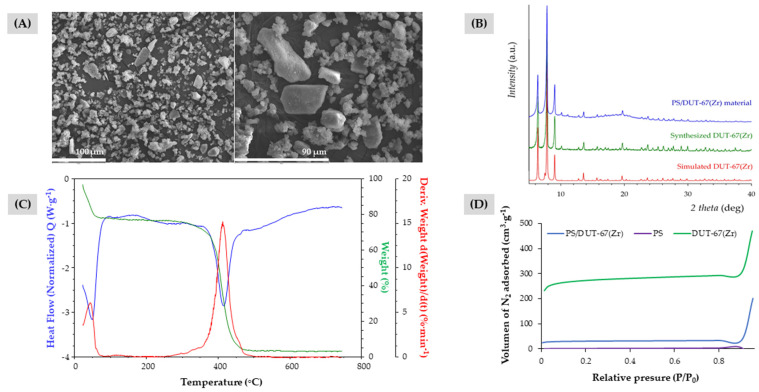
(**A**) SEM images of the PS/DUT-67(Zr) material. (**B**) Powder X-ray diffraction patterns of PS spheres, DUT-67(Zr) and that of the PS/DUT-67(Zr) material. (**C**) TG/DTA analysis of the PS/DUT-67(Zr) material. (**D**) N_2_ isotherm plots of PS spheres, the MOF DUT-67(Zr), and the PS/DUT-67(Zr) material.

Different hybrid materials based on PS and MOFs, such as DUT-67(Zr) and CIM-80(Al), were synthesized by the dispersion polymerization of styrene [32,36]. The utilization of AIBN as initiator, the use of PVP, and the incorporation of MOFs as co-stabilizers, were the key elements for the reaction. The complex covalent interactions established between these components and the monomer have led to the obtention of interesting heterogeneous materials, as it is shown in Figure 2A and in Figures S2 and S3A of the electronic supporting material (ESM).

The successful incorporation of the MOFs in the resulting materials, PS/DUT-67(Zr) and PS/CIM-80(Al), was verified by powder X-ray diffraction (PXRD). PXRD patterns of the hybrid materials and the previously synthesized MOFs as neat materials were compared with the theoretical DUT-67(Zr) and CIM-80(Al) PXRD patterns reported, respectively [37,38]. As it can be observed in Figure 2B) and in Figure S3B, the obtained PXRD patterns agree with the expected ones, supporting the correct formation of the MOFs within the material. In the case of PS/DUT-67(Zr) and PS/CIM-80(Al), the background signal present in their diffraction patterns points to the existence of PS in the hybrid material (PXRD pattern of the neat PS can be observed in Figure 2B).

PS microspheres were also prepared, following the conditions described in Section 2.3.2., and characterized. SEM images (Figure S4A)) show the presence of 1 μm spherical and uniform particles, obtained through the dispersion polymerization method. On the other hand, FT-IR spectrum (Figure S4B)) confirms the PS structural composition based on the absorption peaks at 3021, 1490, 1448, 757, and 694 cm^−1^, corresponding to the phenyl groups, and the peaks at 2910 and 2840 cm^−1^, related to the CH and CH_2_ groups. As highlighted by Yu et al., the absorption peaks at 1594 and 1294 cm^−1^ can be attributed to the stretching vibrations of C=O and C-N bonds, which are characteristics from the pyrrolidone ring of PVP [36].

Due to the experimental limitations of the PS/CIM-80(Al) material when screening it into the analytical application, which will be detailed in Section 3.3., this PS/CIM-80(Al) material was dismissed for additional characterization studies. Thus, further characterization of the PS/DUT-67(Zr) material included the evaluation of its thermal stability by TG/DTA analysis. In Figure 2C, the resulting TG curve shows how the hybrid material experimented a 20% weight loss below 75 °C, possibly assigned to solvent molecules retained on it. After that, there is not any other observable phenomenon up to 300 °C, when the material decomposition starts.

N_2_ adsorption isotherms were also obtained with the aim of calculating the surface area of the hybrid material and its two components: DUT-67(Zr) MOF and PS spheres. Figure 2D shows the isotherm linear plots for all these microporous materials. The resulting BET surface area for the PS/DUT-67(Zr) material was 98.1 m²·g^−1^. This value clearly shows a significant improvement of the nitrogen adsorption if compared to neat PS microspheres, with a BET surface area of 6.6 m²·g^−1^, due to the incorporation of the DUT-67(Zr) MOF (854.7 m²·g^−1^). The same study was carried out for the PS/CIM-80(Al) material and the observed results are similar, the composite exhibits a higher value of the BET surface area (219.1 m^2^·g^−1^) than the PS, due to the inclusion of the CIM-80(Al) (884.0 m^2^·g^−1^) (see Figure S3C).

### 3.2. Chromatographic Method: Analytical Quality Parameters

The chromatographic separation of the six selected PCPs was accomplished in less than 6 min, with proper resolution for all analytes (chromatographic conditions as described in Section 2.2), as shown in Figure S1. Several analytical quality parameters of the UHPLC-UV/Vis method are listed in Table S1. The linear calibration curves were obtained by the direct injection of 20 μL of the calibration standard solutions, reaching coefficients of determination (R^2^) higher than 0.9994. The limits of detection (LODs) and quantification (LOQs) were calculated as 3 and 10 times the relationship between the chromatographic signal and noise (S/N), respectively, and were experimentally verified by the injection of standard solutions at such concentration levels. LODs ranged between 1.50 and 2.00 μg·L^−1^, whereas LOQs ranged from 5.00 to 6.70 μg·L^−1^.

The precision of the UHPLC-UV/Vis method was studied, evaluating the intra-day (*n* = 3) and inter-day (*n* = 9) repeatability at three concentration levels (40, 350, and 3000 μg·L^−1^) over three consecutive days. In both cases, relative standard deviations (RSDs) values were lower than 13.9%.

### 3.3. Preliminary Study: Material Selection

**Figure 3:**
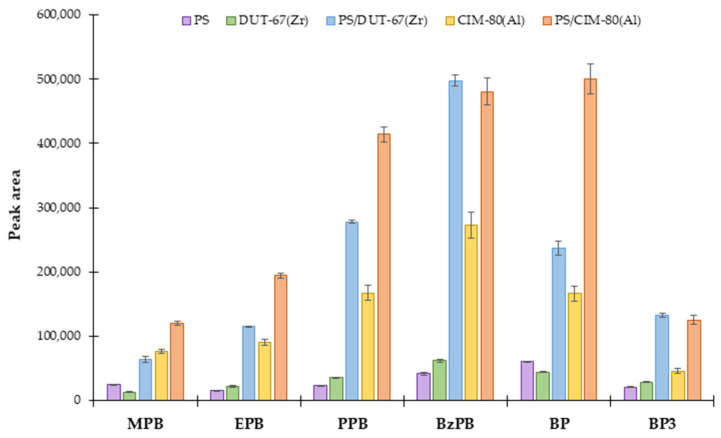
Evaluation of the extraction efficiencies (monitored as peak area of the final chromatographic PCP signals) of different materials used as sorbents in μ-dSPE-UHPLC-UV/Vis with respect to the extraction of PCPs. The materials tested as sorbents included: PS microspheres, neat DUT-67(Zr) MOF, neat CIM-80(Al) MOF, PS/DUT-67(Zr), and PS/CIM-80(Al). Preliminary μ-dSPE conditions included: 10 mg of extraction material as sorbent, 10 mL of an aqueous standard solution of PCPs at a concentration level of 100 μg·L^−1^, 5 min of extraction time under vortex agitation, 150 μL ACN as desorption solvent, and 5 min of desorption time under vortex agitation.

Two different hybrid materials were synthesized and proposed for their potential application for extracting target contaminants (PCPs) from aqueous samples by μ-dSPE-UHPLC-UV/Vis. A comparison study was performed using several preliminary screening conditions: 10 mg of sorbent, 10 mL of an aqueous standard solution of PCPs at a concentration level of 100 μg·L^−1^, 5 min of vortex agitation as extraction time, and as desorption conditions: 150 μL of ACN and 5 min of vortex agitation. Comparison was carried out not only with the hybrid materials prepared, but also with the individual components forming part of the material, because improved performance is pursued (otherwise the material is useless). Figure 3 shows the results of this screening study.

As it can be seen, the hybrid materials (PS/DUT-67(Zr) and PS/CIM-80(Al)) exhibited much higher extraction efficiencies than their unitary components, the PS particles and the corresponding MOF used: DUT-67(Zr) or CIM-80(Al). If only attending to neat PS microspheres, they are able to achieve extraction efficiencies of maximum 3.34%, and therefore, it is clear they are not contributing to the extraction of these target compounds. If comparing only the neat MOFs, CIM-80(Al) showed the best performance. Regarding the resulting hybrid materials, both showed a similar tendency toward the extraction of most of the non-polar analytes, being able to accomplish efficiencies close to 40% for some of them, which is quite adequate for a microextraction method. However, for more polar compounds, like MPB and EPB, the PS/CIM-80(Al) material showed better performance than the PS/DUT-67(Zr) material. These results would justify the logical selection of the hybrid material based on PS and CIM-80(Al) MOF as the extraction sorbent. But, despite its adequate performance, the PS/CIM-80(Al) was dismissed due to the following experimental limitation: higher amounts of the reagents were needed for the synthesis of CIM-80(Al) and, consequently, also high amounts to produce enough PS/CIM-80(Al). This limitation in terms of scalability encouraged us to select the PS/DUT-67(Zr) material.

From an environmental-friendly point of view and considering that CIM-80(Al) MOF has not been successfully scaled yet (on contrary, the scale-up of the DUT-67(Zr) MOF has been demonstrated [35]), the PS/DUT-67(Zr) was selected as the extraction material. In addition, the significant extraction enhancement observed against the PS spheres and the neat MOF made its study more attractive.

Among the interesting features of PS/DUT-67(Zr), it was found out that the practically exponential increase in efficiency of this hybrid materials versus the neat MOF could be caused by its wettability. As it is shown in Figure S5, PS is a hydrophobic material with reduced wettability that involves the formation of an almost perfect water droplet when the material and water get in contact. On the contrary, DUT-67(Zr) is a hydrophilic MOF that get completely wet when it is exposed to an aqueous media. Thus, there is not any droplet observable in its case. As a result, the hydrophobicity of PS/DUT-67(Zr) emerges from these very distinguished properties, associated to the PS spheres and to the neat MOF. This change in the pore environment properties have been already observed in MOF-5-PS particles [39]. In this case, the more hydrophobic environment precludes the adsorption of water molecules and increase the partitioning of target analytes toward the PS/DUT-67(Zr) particles. The resulting PS-MOF synergy led to a hydrophobic material where the pore environment of the MOF is altered by the polystyrene, allowing a better performance than the single (neat) components [40].

### 3.4. Optimization of the μ-dSPE Method

To evaluate if this hydrophobic hybrid PS/DUT-67(Zr) material is an interesting sorbent, valid to perform as an alternative to other sorbents for the monitoring of PCPs using a μ-dSPE-UHPLC-UV/Vis method, different variables were considered in the optimization, such as the sorbent amount, the desorption solvent, and the extraction and desorption times.

**Figure 4:**
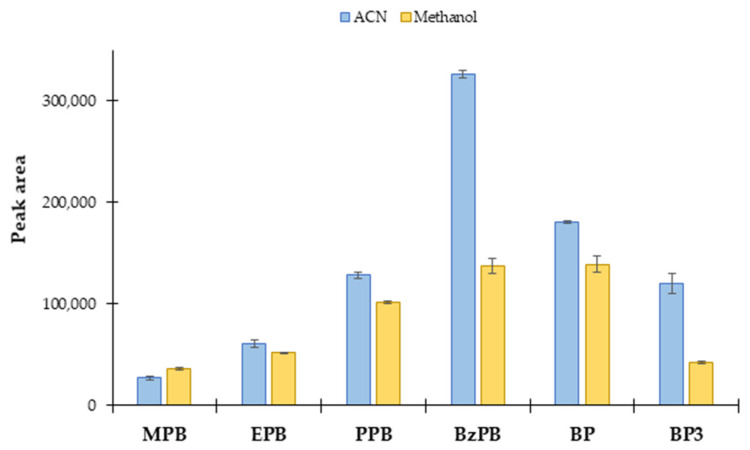
Effect of the desorption solvent in the extraction performance of PCPs by the PS/DUT-67(Zr) material using μ-dSPE. The fixed conditions used were: 10 mg of extraction material, 10 mL of an aqueous standard solution of PCPs at a concentration level of 100 μg·L^−1^, 5 min of extraction time under vortex agitation, 150 μL of desorption solvent, and 5 min of desorption time under vortex agitation.

One of the most relevant factors when using MOFs in dispersive-based microextraction methods is the influence of the desorption solvent, because normally retention of target analytes by the MOF is very strong, and the release is not that straightforward. In this sense, the selection of the best desorption solvent was accomplished following a univariate strategy, by performing extractions in triplicate and using two organic solvents: acetonitrile (ACN) and methanol. The solvents were chosen to ensure avoiding further solvent-exchange steps before UHPLC injection (and therefore simplifying the entire procedure). Several preliminary extraction conditions were fixed in this study: 10 mg of sorbent, 10 mL of an aqueous standard solution of PCPs at a concentration level of 100 μg·L^−1^, 5 min of extraction time under vortex agitation, 150 μL of desorption solvent, and 5 min of desorption time under vortex agitation. Figure 4 shows the results of this study. As it can be seen, the peak areas obtained with ACN as desorption solvent were slightly higher than those obtained with methanol for most of the PCPs, with ACN being particularly efficient for BzPB and BP3. In the case of MPB, the exception for this trend, methanol resulted more favorable for the desorption. For this reason and taking into consideration the use of ACN as one of the mobile phases of the UHPLC-UV/Vis method, ACN was selected as desorption solvent.

**Figure 5:**
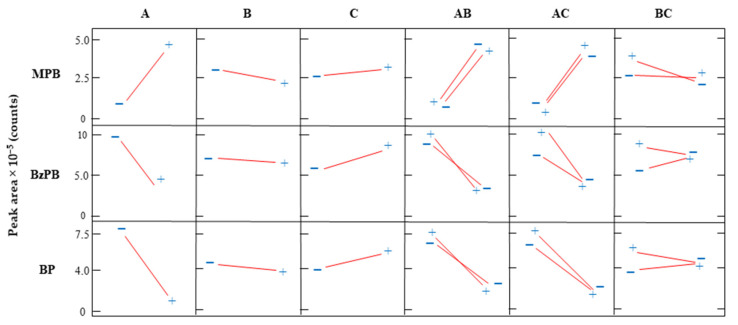
Effect of the main parameters and their interactions in the efficiency of the μ-dSPE-UHPLC-UV/Vis method, based on the chromatographic peak areas of three representatives PCPs (MPB, BzPB, and BP) obtained through the screening analysis. In the graph, A is the amount of sorbent, B is the extraction time, and C is the desorption time.

Once the desorption solvent was set, the remaining parameters that affect the μ-dSPE procedure were optimized. Sample volume was fixed to 10 mL owing to volume limitations of the available centrifuge tubes and the centrifuge characteristics. Experimental variables such as sorbent amount, extraction time, and desorption time, were subjected to a screening design. In this case, a 2*^k^* factorial design (where *k* is the number of studied factors) was applied considering the above-mentioned parameters. A total of eight experiments and three additional replicates were performed. The lowest and highest values selected for each parameter are summarized in Table S2, as well as the conditions applied in each study. Figure 5 shows the effects and interactions of the main factors in the microextraction procedure on three representative PCPs (two preservatives and one UV filter). In general, the amount of sorbent had the strongest and the most compound-dependence influence over the extraction of the PCPs. On the other hand, the time required for an efficient extraction and desorption was not significant. Therefore, based on the screening study, the extraction and desorption time were fixed to the minimum (2 min) and maximum (5 min) value, respectively.

With respect to the amount of sorbent, its effect correlated with the polarity of the studied compounds and their affinities toward the extraction material. This implies the necessity of increasing the amount of PS/DUT-67(Zr) material to improve the extraction efficiencies of those less-extracted compounds, such as MPB, EPB, and PPB, which are also more polar. Meanwhile, better results were obtained with BzPB, BP, and BP3.

As the influence of the amount of sorbent emerged as the most critical, highly analyte-dependent, a secondary univariate study was carried out to test deeply this parameter. In this sense, extractions were performed in duplicate with different amounts of the PS/DUT-67(Zr) material. Figure S6 shows the results of this study. In terms of enrichment factors (E_F_) and extraction efficiencies (E_R_), similar values were obtained if using 20 mg of the sorbent, with differences only noticeable for four PCPs: PPB, BzPB, BP, and BP3. For those compounds, the use of amounts higher than 30 mg of PS/DUT-67(Zr) entailed to a significant extraction efficiency loss (around 35% in some cases). This could be linked to a limitation in the material dispersion and its tendency to form aggregates, affecting its interaction with those analytes. Consequently, the amount of sorbent was set to 20 mg by considering the better extractive response of the material toward the heaviest and more non-polar PCPs and giving them priority on the monitoring. Clearly, this decision is going to affect the efficiency for the most polar PCPs (MPB and EPB particularly).

Finally, the desorption step was also evaluated. In particular, the desorption solvent volume and the number of desorption steps were considered in the optimization process. Three different experiments were performed by changing the volume of ACN and the number of repetitions. As it can be seen in Figure S7A,B, two desorption steps using 200 μL of ACN (100 μL in each) lead to the enhancement of the PCPs preconcentration and allows the obtention of higher E_R_ for all the analytes. Thus, two desorption steps were established as the ideal desorption conditions. Those 200 μL as resulting total volume (coming from the combined 2 desorption steps), was diluted to 600 μL with ultrapure Milli-Q water (1:3 ACN/water ratio) to ensure compatibility with the UHPLC mobile phase composition.

In summary, 20 mg of sorbent, 10 mL of sample, 2 min of extraction time, and two desorption steps of 5 min of desorption time using 100 μL of ACN as desorption solvent in each, were set as the compromise optimum conditions for the monitoring of six PCPs by μ-dSPE-UHPLC-UV/Vis.

### 3.5. Analytical Performance of the μ-dSPE-UHPLC-UV/Vis Method

Several analytical quality parameters were obtained to evaluate the analytical performance of the entire μ-dSPE-UHPLC-UV/Vis procedure. Some of the most relevant results are summarized in Table 2.

Calibration curves were obtained by subjecting aqueous standards (containing the PCPs studied) to the entire μ-dSPE-UHPLC-UV/Vis method. Coefficients of determination (R^2^) higher than 0.9935 were achieved, which are quite adequate considering the influence of the sorbent heterogeneity. LODs and LOQs were estimated as 3 and 10 times the signal-to-noise ratio (S/N), respectively, and then experimentally checked by applying the complete extraction and determination procedure to aqueous standards prepared at those concentration levels. LODs ranged between 0.50 μg·L^−1^ for PPB and 3.00 μg·L^−1^ for MPB, and LOQs ranged between 1.70 μg·L^−1^ for PPB and 10.0 μg·L^−1^ for MPB.

The precision of the entire μ-dSPE-UHPLC-UV/Vis method was evaluated based on the intra-day (*n* = 3) and inter-day (*n* = 9) repeatability at two concentration levels over three consecutive days. The results were expressed as the relative standard deviation (RSD) values at the lower (75 μg·L^−1^) and the higher (650 μg·L^−1^) concentration levels. Intra-day RSD values lower than 18.4 and 19.6%, and inter-day RSD values lower than 23.5 and 21.2% were obtained at the corresponding levels. Inter-batch reproducibility was also assessed by doing extractions in triplicate of aqueous standard solutions at a concentration level of 100 μg·L^−1^ utilizing two sorbent batches synthesized in non-consecutives days. Inter-batch RSD values were lower than 10.9%.

The analytical performance of the proposed extraction procedure, under the optimal conditions, was studied in terms of E_F_ and E_R_. E_F_ values ranged between 0.577 for MPB and 4.94 for BzPB, and E_R_ values ranged between 10.4% for MPB and 89.0% for BzPB were achieved (efficiencies calculated not counting the 1:3 dilution performed before UHPLC injection, to show the performance of the microextraction approach).

### 3.6. Comparison with Other Methods

The analytical performance of the proposed μ-dSPE-UHPLC-UV/Vis method was compared with other methods reported in the literature for the monitoring of PCPs in samples of different nature, as shown in Table S3 of the ESM [41,42,43,44,45,46,47,48,49,50].

From the table, it can be observed that the main advantages of the developed procedure relate to the short extraction times and the relatively small amount of PS/DUT-67(Zr) required for the proper extraction of PCPs. This cannot be only attributed to the properties of the synthesized material but also to the SPE technique selected for it. In terms of precision, despite of being the method with the highest maximum RSD value reported, this may be considered as adequate if the structure of the hybrid material is taking into account. Moreover, quite similar RSDs values can be obtained when neat MOFs [47] or MOFs-based composites [50] are used as sorbents, among other materials [43].

In the case of the sensitivity, the proposed μ-dSPE-UHPLC-UV/Vis method presents good LODs with respect to the last four PCPs studied (PPB, BzPB, BP and BP3), the ones with a better affinity toward the extraction material. It must be also considered that UV/Vis detection and not MS has been employed in this study.

### 3.7. Analysis of Micellar Cosmetic Samples

Three commercial micellar cosmetic samples, labelled as 1, 2, and 3, were analyzed with the previously optimized and validated μ-dSPE-UHPLC-UV/Vis method. None of the six PCPs were detected in the samples, as they were claimed as PCP free. Possible matrix effects caused by the nature of the samples were verified by performing recoveries studies with samples spiked at a concentration level of 75 μg·L^−1^. Table S4 summarizes the obtained relative recoveries (RR) for the different analytes in each sample.

Positive matrix effects were observed for all PCPs with the exceptions of BP3 in sample 1, BP in sample 2, and EPB, PPB, and BP in sample 3. As it is indicated in Table S4, MPB could not be correctly determined due to some sample interference. For the proper monitoring of these compounds in this kind of samples, calibrations in the matrix or a standard addition method would be required.

## 4. Conclusions

Novel hybrid materials based on PS and MOFs, such as DUT-67(Zr) and CIM-80(Al), were successfully synthesized by dispersion polymerization of styrene. The resulting materials, PS/DUT-67(Zr) and PS/CIM-80(Al), were successfully prepared and characterized, while being evaluated for their potential application as sorbent in μ-dSPE. These composites demonstrated much higher extraction efficiencies toward a group of PCPs studied than their unitary components, standing out for the extraction and preconcentration of the most non-polar ones. Considering the synthetic limitations related to PS/CIM-80(Al), PS/DUT-67(Zr) was selected and incorporated as the adequate sorbent for the determination of six PCPs in micellar cosmetics products by μ-dSPE in combination with UHPLC-UV/Vis. The entire μ-dSPE-UHPLC-UV/Vis method was properly optimized through univariate studies and a 2*^k^* factorial design.

Owing to its outstanding hydrophobicity, the PS/DUT-67(Zr) exhibited an exponential enhancement of the extraction capacity in comparison with its neat single components, while performing adequately in an analytical microextraction dispersive method combined with UHPLC. Ongoing work is aimed to study more deeply the structure of these materials and the interactions set between their compositional elements, as well as with the proposed PCPs. The impact of this and other highly hydrophobic materials prepared with this procedure is also under study.

## Figures and Tables

**Table 1:** Structure and several physicochemical properties of the analytes selected (taken from PubChem^®^, 2021).

Analyte (Abbreviation)	Structure	Molecular Weight (g·mol^−1^)	Molecular Dimensions ^a^ (Å^3^)	Vapor Pressure ^b^ (N·m^−2^)	pKa	Log K_ow_^c^
Methylparaben (MPB)	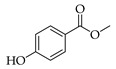	152.15	5 × 3 × 9	3.16×10^−2^	8.50	1.96
Ethylparaben (EPB)	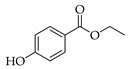	166.17	5 × 4 × 11	1.24×10^−2^	8.34	2.47
Propylparaben (PPB)	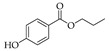	180.20	5 × 4 × 12	4.09×10^−2^	8.50	3.04
Benzylparaben (BzPB)	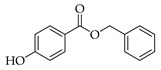	228.24	9 × 8 × 12	-	-	3.56
Benzophenone (BP)	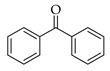	182.22	6 × 6 × 9	0.257	-	3.18
Benzophenone-3 (BP3)	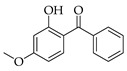	228.24	6 × 6 × 12	8.83×10^−4^	7.10	3.79

^a^ Approximate molecular dimension, estimated from the simulated structure, and expressed as height × depth × length (h × d × l). ^b^ At 25 °C. ^c^ Octanol-water partition coefficient.

**Table 2:** Analytical quality parameters of the proposed μ-dSPE-UHPLC-UV/Vis method.

PCPs	Slope ± S_b_^a^	R^2 b^	S_x/y_^c^	LOD ^d^ (μg·L^−1^)	Working Range (μg·L^−1^)	Intra-Day RSD ^e^ (%)		Inter-Day RSD ^e^ (%)		E_F_ ^f^	E_R_ ^g^ (%)
						75 (μg·L^−1^)	650 (μg·L^−1^)	75 (μg·L^−1^)	650 (μg·L^−1^)		
MPB	972 ± 23	0.9978	18,182	3.00	10.0–800	8.29	13.6	21.8	20.4	0.577	10.4
EPB	1425 ± 28	0.9985	21,875	1.30	4.30–800	7.50	12.5	21.5	21.2	1.19	21.4
PPB	3001 ± 41	0.9993	32,088	0.50	1.70–800	5.72	10.5	20.4	21.1	2.28	41.1
BzPB	4868 ± 176	0.9935	139,562	0.75	2.50–800	3.21	8.77	10.3	14.8	4.94	89.0
BP	3626 ± 62	0.9988	48,736	1.00	3.35–800	18.4	10.8	23.5	13.3	2.52	45.4
BP3	969 ± 11	0.9995	8575	1.00	3.35–800	12.2	19.6	12.5	12.9	1.96	35.3

^a^ Standard deviation related to the slope. ^b^ Coefficient of determination. ^c^ Standard deviation of the residuals (or error of the estimate). ^d^ Limit of detection. ^e^ Relative standard deviation expressed as %: intra-day (*n* = 3) and inter-day (*n* = 9 in three consecutive days) for each specified concentration level. ^f^ Enrichment factor estimated by the slopes of the UHPLC-UV/Vis method and μ-dSPE-UHPLC-UV/Vis method. ^g^ Extraction efficiency estimated by the slopes of the UHPLC-UV/Vis method and μ-dSPE-UHPLC-UV/Vis method (not counting the 1:3 dilution before UHPLC injection).

## Data Availability

The data presented in this study are available in supplementary material.

## Supplementary Materials

The following are available online. Figure S1: Representative chromatogram (UHPLC-UV/Vis), under optimum conditions, obtained by the direct injection of a standard solution of six PCPs at a concentration level of 100 μg·L^−1^, Figure S2: SEM images of the PS/DUT-67(Zr) material at higher magnifications, Figure S3: (A) SEM images of the PS/CIM-80(Al) material. (B) Powder X-ray diffraction patterns of CIM(Al) and that of the PS/CIM-80(Al) material. (C) N_2_ isotherm plots of PS spheres, the MOF CIM-80(Al), and the PS/CIM-80(Al) material, Figure S4: (A) SEM images of PS microspheres. (B) Fourier transform infrared (FT-IR) spectrum of PS microspheres, Figure S5: Wettability of the PS/DUT-67(Zr) hybrid material (bottom and up middle) compared to that of the neat components: DUT-67(Zr), on the upper left, and PS microspheres, on the upper right, Figure S6: Effect of the amount of the PS/DUT-67(Zr) material in the extraction of six PCPs by μ-dSPE, under the following fixed conditions: 10 mL of an aqueous standard solution of PCPs at a concentration level of 100 μg·L^−1^, 2 min of extraction time under vortex agitation, 150 μL ACN as desorption solvent, and 5 min of desorption time under vortex agitation. Extraction efficiencies values are calculated for the µ-dSPE method without counting the further 1:3 dilution before UHPLC injection, Figure S7: Enrichment factors (A) and extraction efficiencies (B, not counting the further 1:3 dilution) values, depending on the desorption solvent volume and the number of desorption steps, evaluated in the optimization of the desorption procedure. The fixed μ-dSPE conditions were: 20 mg of extraction material, 10 mL of an aqueous standard solution at a concentration level of 100 μg·L^−1^, 2 min of extraction time under vortex agitation, and 5 min of vortex agitation as desorption time. Experiments were performed in triplicate. Table S1: Several analytical quality parameters of the UHPLC-UV/Vis method, Table S2: Experimental conditions considered in the screening design for the optimization of the three parameters selected: amount of sorbent, extraction time, and desorption time, together with the set of experiments required, Table S3: Comparison with other methods reported in the literature for the monitoring of PCPs using sorbent-based approaches and liquid chromatography, Table S4: Recoveries study in micellar cosmetic samples (spiked at 75 μg·L^−1^) using the μ-dSPE-UHPLC-UV/Vis method proposed.

## Sample Availability

Samples of the compounds are not available from the authors.

## Abbreviations

ACN	Acetonitrile
AIBN	Azobisisobutyronitrile
BET	Brunauer, Emmett and Teller
BP	Benzophenone
BP3	Benzophenone-3
BzPB	Benzylparaben
CIM	Canary Islands Materials (code for MOFs)
COFs	Covalent-organic frameworks
DESs	Deep eutectic solvents
DMF	Dimethylformamide
DUT	Dresden University of Technology (code for MOFs)
E_F_	Enrichment factor
EPB	Ethylparaben
E_R_	Extraction efficiency
ESM	Electronic supporting material
FT-IR	Fourier transform infrared spectroscopy
H_2_TDC	2,5-thiophenedicarboxylic acid
ILs	Ionic liquids
LC-MS	Liquid chromatography tandem mass spectrometry
LLE	Liquid-liquid extraction
LODs	Limits of detection
LOQs	Limits of quantification
MIL	Material Institute Lavoisier (code for MOFs)
MIPs	Molecular imprinted polymers
MOFs	Metal-organic frameworks
MPB	Methylparaben
NPs	Nanoparticles
PCPs	Personal care products
PPB	Propylparaben
PS	Polystyrene
PS/CIM-80(Al)	Polystyrene/CIM-80(Al) MOF-based hybrid material
PS/DUT-67(Zr)	Polystyrene/DUT-67(Zr) MOF-based hybrid material
PS/MOF	Hybrid material based on polystyrene and metal-organic framework
PVDF	Polyvinylidene fluoride
PVP	Polyvinylpyrrolidone
PXRD	Powder X-ray diffraction
R^2^	Coefficient of determination
RR	Relative recoveries
RSDs	Relative standard deviations
S/N	Signal-to-noise ratio
SBUs	Secondary building units
SEMs	Scanning electron microscopes
SPE	Solid-phase extraction
SPME	Solid-phase microextraction
TG/DTA	Thermogravimetric/differential thermal analysis
UHPLC	Ultra-high performance liquid chromatography
UHPLC-UV/Vis	Ultra-high performance liquid chromatography coupled to ultraviolet/visible detection
UV/Vis	Ultraviolet/visible detection
ZIF	Zeolitic imidazolate framework (code for MOFs)
μ-dSPE	Dispersive micro-solid-phase extraction
μ-SPE	Micro-solid-phase extraction
