# Supplementary material for: Hybrid Materials Formed with Green Metal-Organic Frameworks and Polystyrene as Sorbents in Dispersive Micro-Solid-Phase Extraction for Determining Personal Care Products in Micellar Cosmetics
Source: Molecules. 2022 Jan 26;27(3):813. doi: 10.3390/molecules27030813 (PMC8838677; doi:10.3390/molecules27030813)
Supplement: Supplementary file 1 [file molecules-27-00813-s001.zip › molecules-1555290-supplementary.pdf]

Electronic Supporting Material

Hybrid materials formed with green metal-organic frameworks and polystyrene as sorbents in dispersive micro-solid-phase extraction for determining personal care products in micellar cosmetics

Patricia I. Napolitano-Tabares[1], Adrián Gutiérrez-Serpa[1, 2], Ana I. Jiménez-Abizanda[1], Francisco Jiménez-Moreno[1], Jorge Pasán[3] and Verónica Pino[1, 2]

[1]Laboratorio de Materiales para Análisis Químicos (MAT4LL), Departamento de Química, Unidad Departamental de Química Analítica, Universidad de La Laguna (ULL), La Laguna, Tenerife 38206, Spain

[2]Unidad de Investigación de Bioanalítica y Medioambiente, Instituto Universitario de Enfermedades Tropicales y Salud Pública de Canarias, Universidad de La Laguna (ULL), 38206 Tenerife, Spain.

[3]Laboratorio de Materiales para Análisis Químicos (MAT4LL), Departamento de Química, Unidad Departamental de Química Inorgánica, Universidad de La Laguna (ULL), La Laguna, Tenerife 38206, Spain.

Table of Contents:

Figure S1 1

Figure S2 2

Figure S3 3

Figure S4 4

Figure S5 5

Figure S6 6

Figure S7 7

Table S1 8

Table S2 9

Table S3 10

Table S4 12

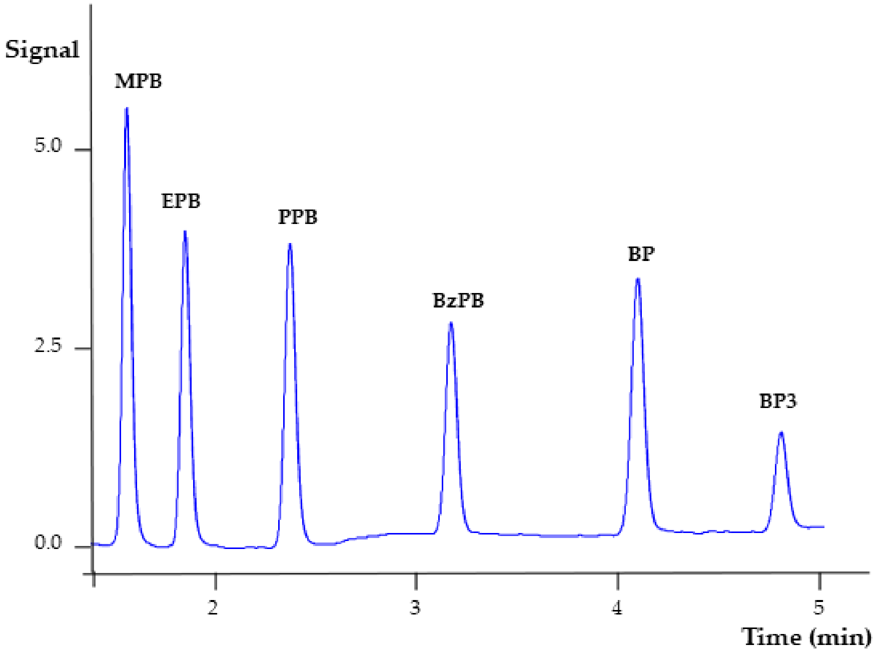

Figure S1. Representative chromatogram (UHPLC-UV/Vis), under optimum conditions, obtained by the direct injection of a standard solution of six PCPs at a concentration level of 100 μg·L^{-1}.

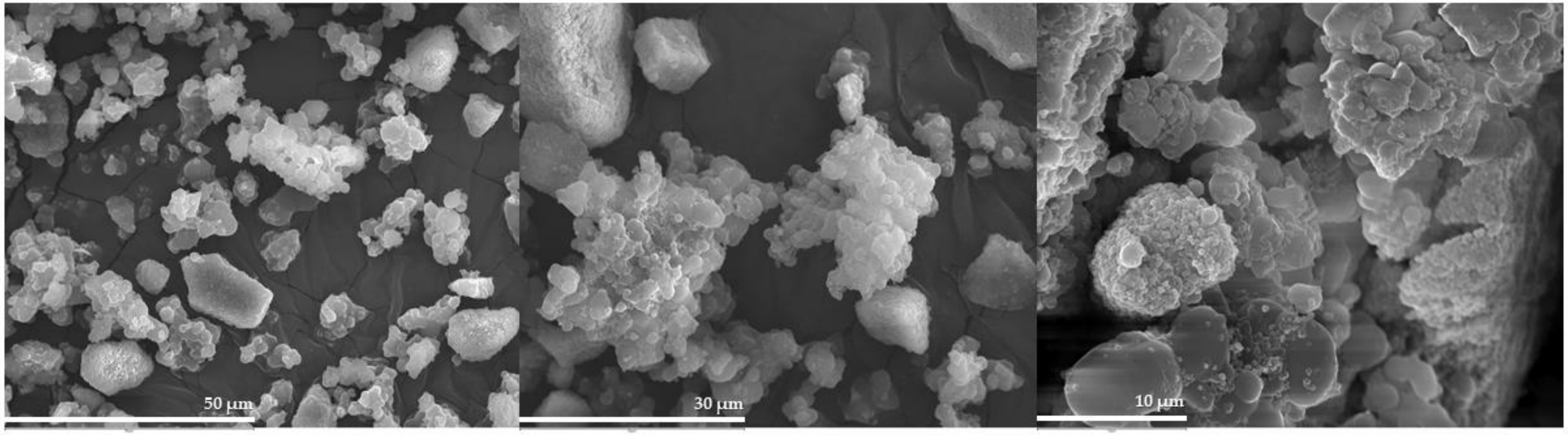

Figure S2. SEM images of the PS/DUT-67(Zr) material at higher magnifications.

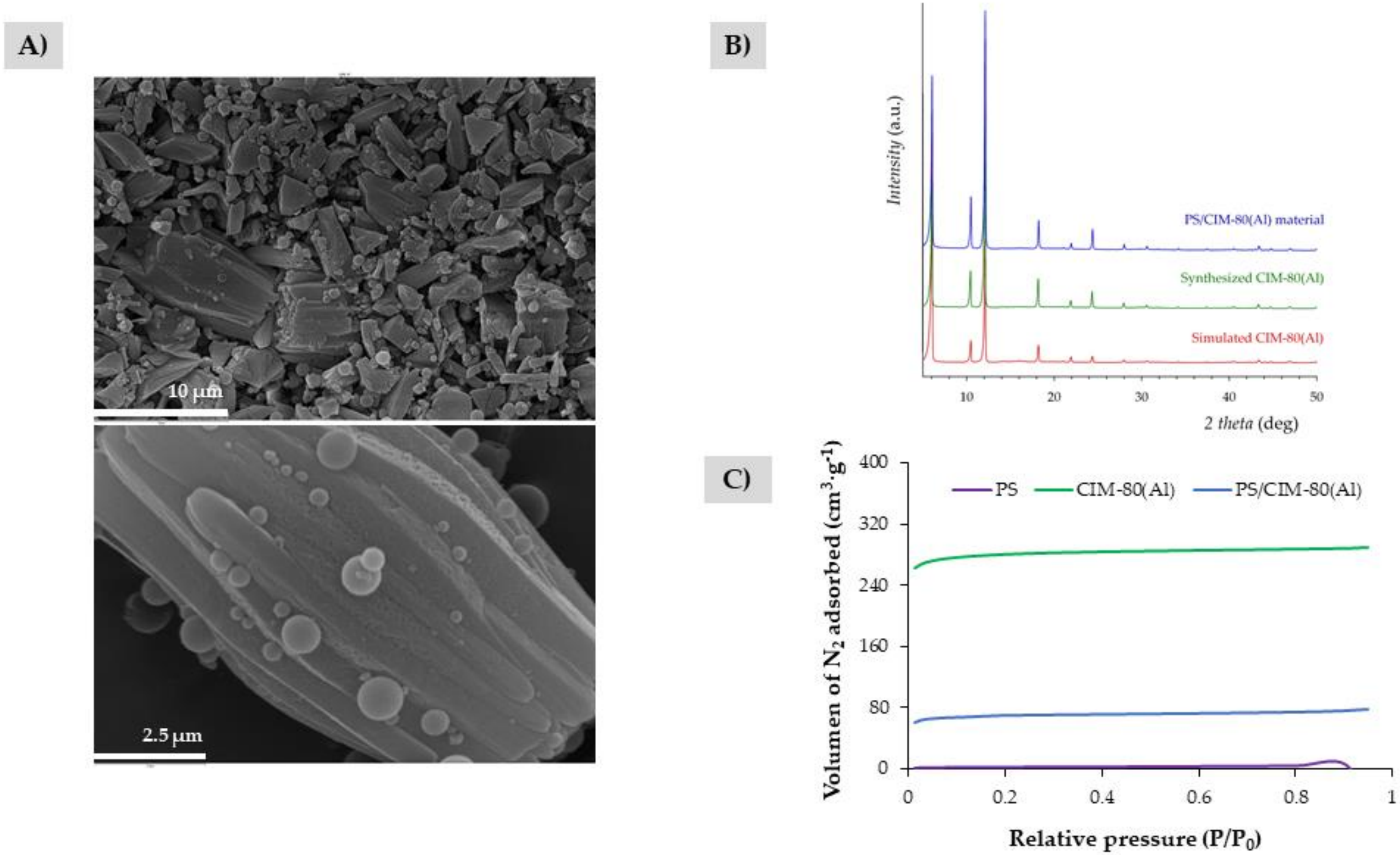

Figure S3. A) SEM images of the PS/CIM-80(Al) material. **B)** Powder X-ray diffraction patterns of CIM(Al) and that of the PS/CIM-80(Al) material. **C)** N_2 isotherm plots of PS spheres, the MOF CIM-80(Al), and the PS/CIM-80(Al) material.

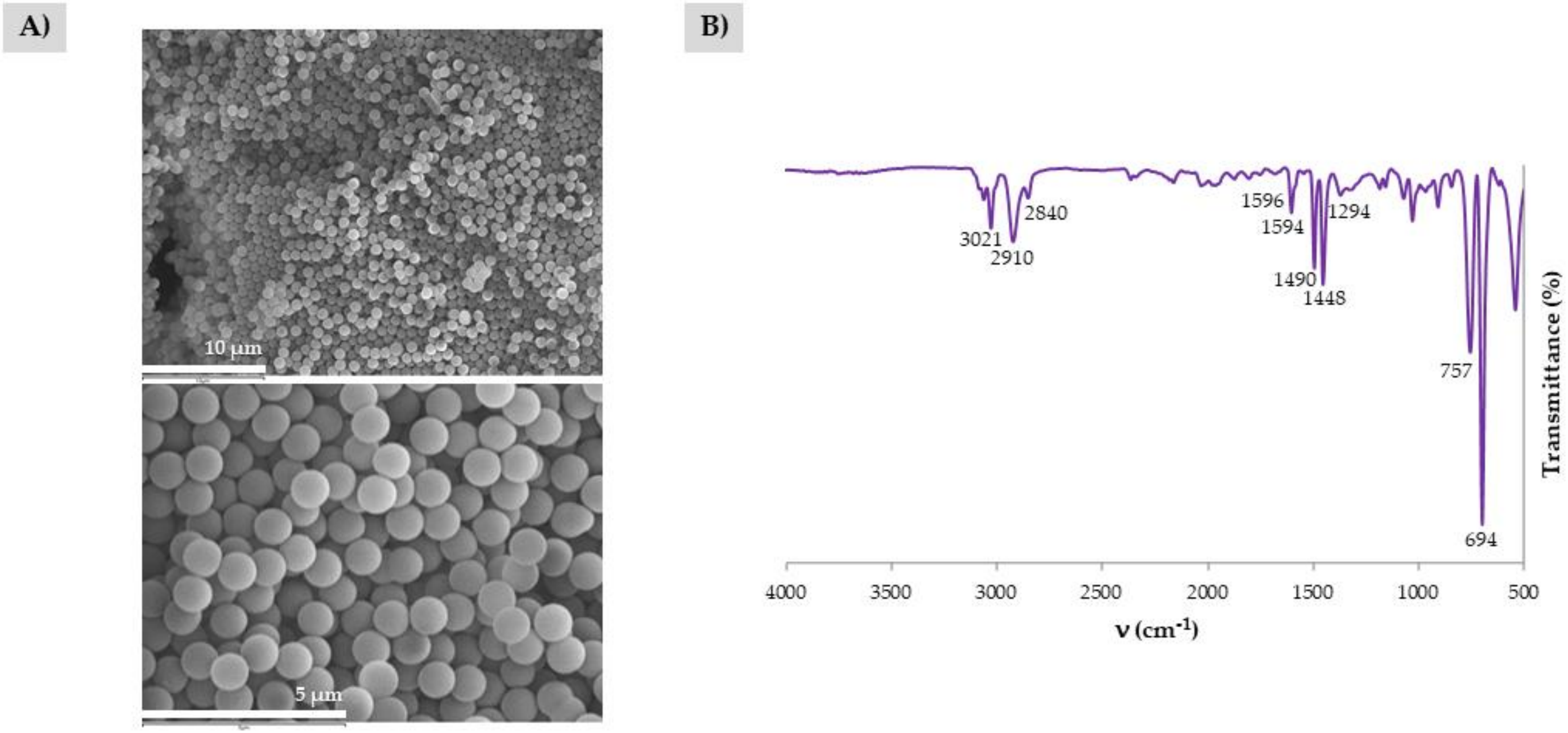

Figure S4. A) SEM images of PS microspheres. **B)** Fourier transform infrared (FT-IR) spectrum of PS microspheres.

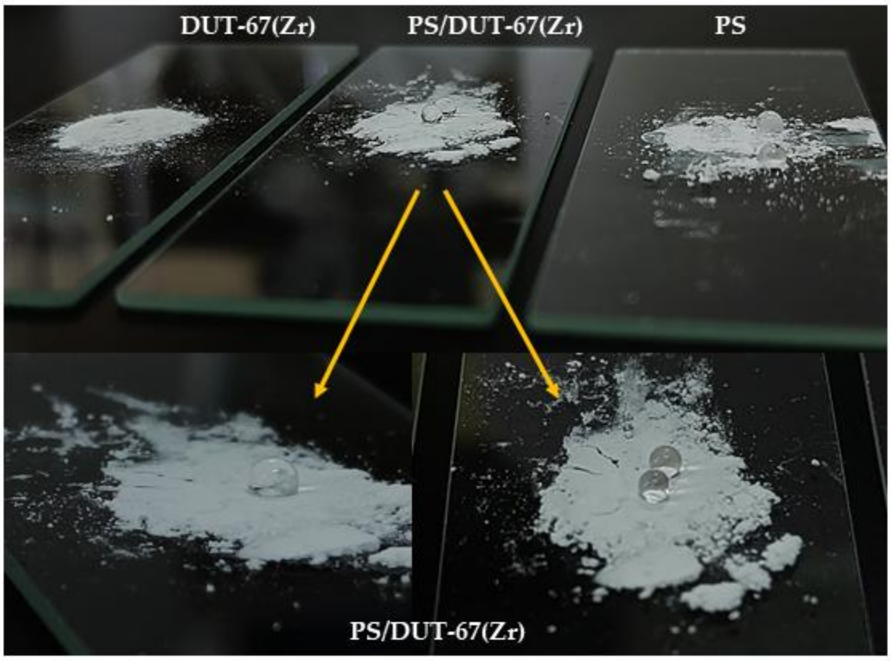

Figure S5. Wettability of the PS/DUT-67(Zr) hybrid material (bottom and up middle) compared to that of the neat components: DUT-67(Zr), on the upper left, and PS microspheres, on the upper right.

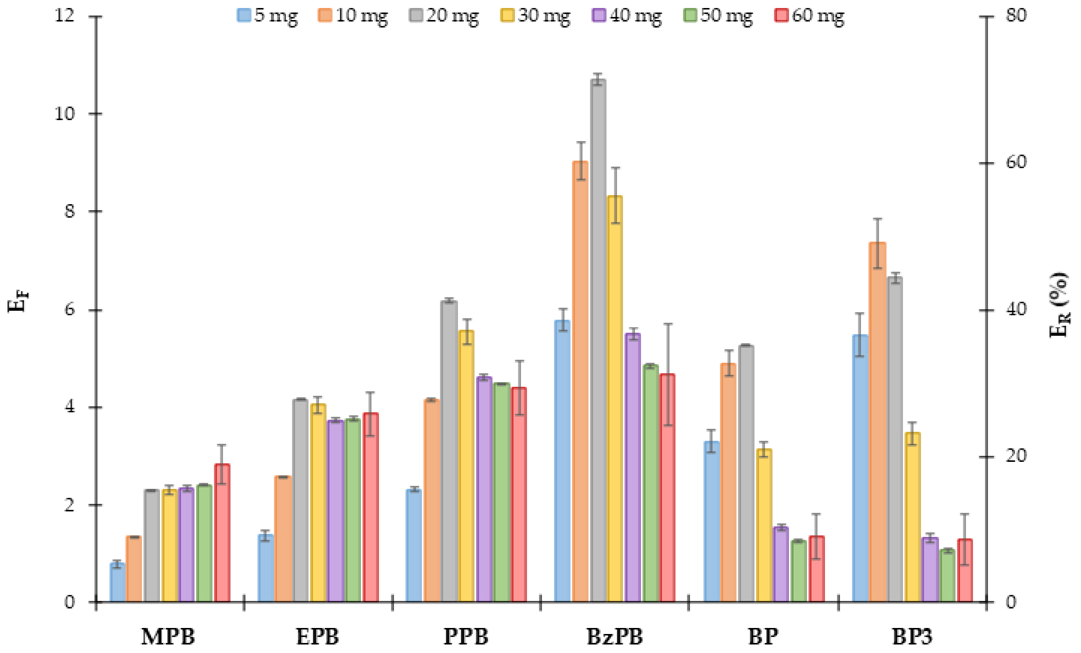

Figure S6. Effect of the amount of the PS/DUT-67(Zr) material in the extraction of six PCPs by μ-dSPE, under the following fixed conditions: 10 mL of an aqueous standard solution of PCPs at a concentration level of 100 μg·L^{-1}, 2 min of extraction time under vortex agitation, 150 μL ACN as desorption solvent, and 5 min of desorption time under vortex agitation. Extraction efficiencies values are calculated for the μ-dSPE method without counting the further 1:3 dilution before UHPLC injection.

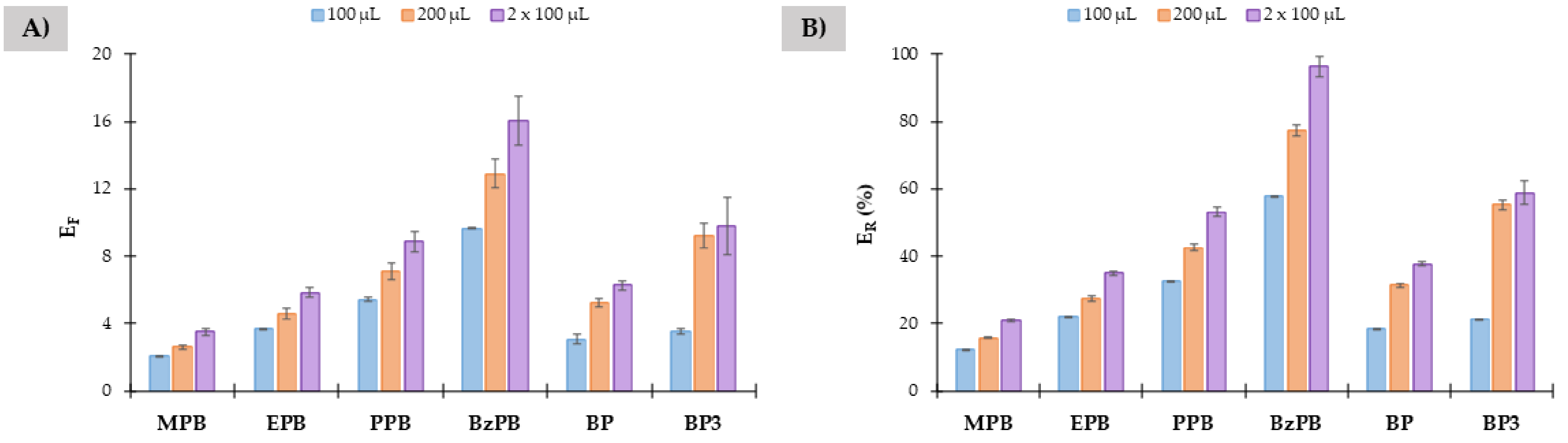

Figure S7. Enrichment factors (**A**) and extraction efficiencies (**B**, not counting the further 1:3 dilution) values, depending on the desorption solvent volume and the number of desorption steps, evaluated in the optimization of the desorption procedure. The fixed µ-dSPE conditions were: 20 mg of extraction material, 10 mL of an aqueous standard solution at a concentration level of 100 µg·L^{-1}, 2 min of extraction time under vortex agitation, and 5 min of vortex agitation as desorption time. Experiments were performed in triplicate.

Table S1. Several analytical quality parameters of the UHPLC-UV/Vis method.

PCP	Retention times ± SD [a]	Slope ± S_b [b]	R^2 [c]	$S_{x/y}$ [d]	LOD [e] (µg·L^{-1})	Working range (µg·L^{-1})	Intra-day RSD [f] (%)			Inter-day RSD [f] (%)		
							40 µg·L^{-1}	350 µg·L^{-1}	3 mg·L^{-1}	40 µg·L^{-1}	350 µg·L^{-1}	3 mg·L^{-1}
MPB	1.705 ± 0.004	1684 ± 14	0.9994	87865	1.70	5.70 – 6000	2.75	0.342	0.565	5.07	2.50	3.07
EPB	2.115 ± 0.006	1201 ± 9	0.9996	55282	1.50	5.00 – 6000	2.91	0.161	0.592	3.36	2.81	3.03
PPB	2.851 ± 0.010	1315 ± 11	0.9995	67711	1.50	5.00 – 6000	4.89	0.301	0.641	4.67	2.94	2.97
BzPB	3.976 ± 0.014	985 ± 6	0.9997	35270	2.00	6.70 – 6000	3.12	0.343	1.03	4.31	3.28	3.03
BP	5.259 ± 0.013	1438 ± 8	0.9998	49287	2.00	6.70 – 6000	0.781	0.532	1.05	2.01	2.59	2.97
BP3	6.247 ± 0.012	494 ± 3	0.9997	18645	1.50	5.00 – 6000	13.9	1.18	0.943	12.1	3.24	2.61

[a] Standard deviation of the retention time (n = 30).

[b] Standard deviation of the slope.

[c] Coefficient of determination.

[d] Standard deviation of the residuals (or error of the estimate).

[e] Limit of detection.

[f] Relative standard deviation expressed as %: intra-day (n = 3) and inter-day (n = 9 in three consecutive days), for each concentration level.

Table S2. Experimental conditions considered in the screening design for the optimization of the three parameters selected: amount of sorbent, extraction time, and desorption time, together with the set of experiments required.

	**Amount of sorbent (mg)**	**Extraction time (min)**	**Desorption time (min)**
Minimum (-)	10	2.0	2.0
Maximum (+)	50	5.0	5.0
		Screening study experiments	
Experiment 1	+	-	+
Experiment 2	-	-	-
Experiment 3	*	*	*
Experiment 4	*	*	*
Experiment 5	*	*	*
Experiment 6	-	+	+
Experiment 7	-	+	-
Experiment 8	-	-	+
Experiment 9	+	-	-
Experiment 10	+	+	-
Experiment 11	+	+	+

* Additional experiments with the intermediate experimental conditions: 30 mg of sorbent and 3.5 min for both the extraction and the desorption time.

Table S3. Comparison with other methods reported in the literature for the monitoring of PCPs using sorbent-based approaches and liquid chromatography.

Sorbent [a]	Analytical method [b]	PCPs (number)	Sample (Volume, mL)	Amount of sorbent (mg)	Extraction time (min)	LODs (µg·L^{-1})	RSD$_{max}$ (%) * (Spiked level)	Reference
CS-Ni/Fe-LDH	µ-SPE-HPLC-PDA	parabens (4)	cosmetics diluted and methanolic extracts of cosmetics diluted (100)	60	100	0.005 - 0.020	2.99 (-)	[41]
MPC@Al_2O_3-SiO_2	µ-dSPE-HPLC-DAD	antimicrobials, UV-filters, and insect repellents (3)	waters (1)	17	20	0.06 - 0.096	4.4 (-)	[42]
Cork	µ-SPE-HPLC-DAD	parabens and UV-filters (5)	waters (0.8)	5.0	1.5	0.6 - 1.4	23 (150 µg·L^{-1})	[43]
MIL-68(Al)-MeS	µ-dSPE-HPLC-UV/Vis	parabens (4)	cosmetics dissolved in ACN and diluted (5)	-	12	0.26 - 0.41	13.8 (40 µg·L^{-1}) **	[44]
CIM-81(Zn)	µ-dSPE-UHPLC-UV/Vis	parabens, UV-filters, and disinfectant (9)	waters (10)	10	1	0.5 - 1.5	13 (15 µg·L^{-1})	[45]
CNDs	µ-SPE-UHPLC-MS/MS	parabens (3)	waters (50)	170	50	0.075 - 0.125	4.09 (50 µg·L^{-1}) **	[46]
HKUST-1(Cu)	µ-dSPE-HPLC-DAD	parabens (7)	waters, urine diluted, and methanolic extracts of cosmetics diluted (20)	150	5	0.1 - 0.6	16 (7 µg·L^{-1})	[47]
Fe_3O_4/GO	m-µ-dSPE-HPLC-DAD	parabens and UV-filters (5)	waters (30)	25	15	0.02 - 0.18	7.6 (-) **	[48]

CS-AC	µ-SPE-HPLC-PDA	parabens (4)	waters (-)	81	~ 1	0.006 - 0.015	3.9 (50 µg·L^{-1})	[49]
PVDF/DUT-52(Zr) MMM	TFME-UHPLC-UV/Vis	parabens and UV-filters (6)	ethanolic extracts of cosmetics diluted (20)	-	90	0.02 - 0.7	23 (100 µg·L^{-1}) **	[50]
PS/DUT-67(Zr)	µ-dSPE-UHPLC-UV/Vis	parabens and UV-filters (6)	cosmetics diluted (10)	20	2	0.50 - 3.0	23.5 (75 µg·L^{-1})	This study

* RSD_{max} as maximum relative standard deviation reported based on the PCPs studied (in %).

** Intra-day precision.

[a] Abbreviations for the sorbents: CNDs for carbon nanodots; CS-AC for chitosan-coated activated carbon composite; CS-Ni/Fe-LDH for chitosan-Ni/Fe-layered double hydroxide composite; Fe_3O_4/GO for magnetite nanoparticles/graphene oxide composite; MIL-68(Al)-MeS for MOF MIL-68(Al)/melamine sponge composite; MPC@Al_2O_3-SiO_2 for composite based on mesoporous carbon, alumina, and silica; PVDF/DUT-52(Zr) MMM for polyvinylidene fluoride/DUT-52(Zr) MOF mixed-matrix membrane.

[b] Abbreviations for the analytical method: DAD for diode array detector; HPLC for high performance liquid chromatography; m-µ-dSPE for magnetic µ-dSPE; MS/MS for tandem mass spectrometry; PDA for photodiode array detector; TFME for thin-film microextraction.

For the definition of the abbreviations not included here, please refer to the list of abbreviations at the end of the article.

Table S4. Recoveries study in micellar cosmetic samples (spiked at 75 μg·L^{-1}) using the μ-dSPE-UHPLC-UV/Vis method proposed.

PCP	Sample 1		Sample 2		Sample 3	
	RR [a] (%)	RSD [b] (%)	RR [a] (%)	RSD [b] (%)	RR [a] (%)	RSD [b] (%)
MPB	194	19	*	*	*	*
EPB	176	20	151	6.8	115	18
PPB	163	16	149	6.2	120	17
BzPB	124	9.7	139	8.0	130	12
BP	151	10	111	12	96.0	12
BP3	111	3.5	137	21	149	4.4

[a] Relative recovery at a spiked concentration level of 75 μg·L^{-1} (n = 3, intra-day).
[b] Relative standard deviation, as %.
* Presence of interferences due to the sample nature.
